# The Effects of AHCC®, a Standardized Extract of Cultured *Lentinura edodes* Mycelia, on Natural Killer and T Cells in Health and Disease: Reviews on Human and Animal Studies

**DOI:** 10.1155/2019/3758576

**Published:** 2019-12-20

**Authors:** Min Sun Shin, Hong-Jai Park, Takahiro Maeda, Hiroshi Nishioka, Hajime Fujii, Insoo Kang

**Affiliations:** ^1^Department of Internal Medicine, Yale University School of Medicine, New Haven, CT 06520, USA; ^2^Research and Development Division, Amino Up Co., Ltd., 004-0839 Sapporo, Japan

## Abstract

Mushrooms have been used for various health conditions for many years by traditional medicines practiced in different regions of the world although the exact effects of mushroom extracts on the immune system are not fully understood. AHCC® is a standardized extract of cultured shiitake or *Lentinula edodes* mycelia (ECLM) which contains a mixture of nutrients including oligosaccharides, amino acids, and minerals obtained through liquid culture. AHCC® is reported to modulate the numbers and functions of immune cells including natural killer (NK) and T cells which play important roles in host defense, suggesting the possible implication of its supplementation in defending the host against infections and malignancies via modulating the immune system. Here, we review *in vivo* and *in vitro* effects of AHCC® on NK and T cells of humans and animals in health and disease, providing a platform for the better understanding of immune-mediated mechanisms and clinical implications of AHCC®.

## 1. Introduction

Mushrooms have been considered to have possible beneficial effects in health and disease for many years by traditional medicines practiced in different regions of the world [1]. Although the exact biological mechanisms underlying such effects are yet to be elucidated, extracts from a group of mushrooms are now used as dietary supplements and functional foods in health conditions possibly associated with immune dysregulations that include infections, inflammatory diseases, and malignancies [1]. The effects of mushrooms on the immune system could stem from bioactive polysaccharides such as beta- (*β*-) glucans or polysaccharide complexes in mushrooms in that these molecules appear to affect innate and adaptive immune responses [2, 3]. Also, studies reported the activation of natural killer (NK) and T cells by alpha- (*α*-) glucans extracted from edible mushrooms like *Tricholoma matsutake* and maitake (*Grifola frondosa*) [4, 5], supporting the implication of *α*-glucans in regulating the immune system.

AHCC® is a standardized extract of cultured shiitake or *Lentinula edodes* mycelia (AHCC®) which contains a mixture of nutrients including oligosaccharides, amino acids, and minerals obtained through the liquid culture process of shiitake mycelia [6, 7]. It is produced by Amino Up Co., Ltd. (Sapporo, Japan) under the trademark “AHCC®.” Hereinafter, AHCC® and ECLM are used interchangeably in the manuscript. The shiitake mycelia used for AHCC® are cultured in a liquid medium where the mycelia proliferate and form globular fungal bodies but not fruiting bodies [8]. AHCC® is produced through the unique manufacturing process of culturing the mycelia followed by separation, sterilization, and freeze-drying [8]. The most abundant component of AHCC® is oligosaccharides which comprise about 74% of the dry weight of AHCC® [6, 7]. Of the oligosaccharides in AHCC®, about 20% are *α*-1,4-glucans, of which a proportion is partially acylated, with a mean molecular weight around 5000 Daltons [6, 7]. The effects of AHCC® on immune cells of humans and animals were reported in *in vitro* and *in vivo* studies, suggesting the possible help of its supplementation in defending the host against infections and malignancies via modulating the immune system [6, 9–28]. This review focuses on the reported effects of AHCC® on natural killer (NK) and T cells given their roles in host defense and inflammation [29–34], providing a platform for the better understanding of immune-mediated mechanisms and clinical implications of AHCC® and possibly other medical mushrooms in health and disease.

## 2. Effects of AHCC® on Natural Killer (NK) Cells in Infections and Malignancies

NK cells are large granular lymphocytes considered as the first line of defense against viral infections and possibly malignancies via secreting cytokines and expressing cytotoxic molecules [30, 34, 35]. Indeed, NK cells are armed with receptors that sense signals from target cells such as infected or tumorous cells, leading to killing [31, 34]. Impaired function or deficiency of NK cells has been associated with increased risk of infections and malignancies in humans and animals [34, 35]. Mushroom products have been suggested to modulate NK cell activity against infected or tumorous cells [36]. A recent study showed that water and ethanol extracts of cultured mycelium from various species could have distinct effects on NK cell-mediated cytotoxity against tumor cells [37]. Water extracts of cultured mycelium from medicinal mushrooms including *Agaricus blazei* and *Ganoderma lucidum* enhanced cytotoxic activity in human NK cell lines by upregulating the cytotoxic molecules perforin and granulysin as well as the NK cell receptors natural killer group 2D (NKG2D) and natural cytotoxicity receptors (NCR) [37]. However, ethanol extracts of the mycelium from the same mushrooms inhibited the expression of these molecules by the same NK cells [37]. These findings support the notion that the mode of extraction of medicinal mushrooms may influence the immunomodulatory effects of the mushrooms on NK cells [37]. The possible effects of AHCC® on NK cells of humans and mice were reported in different clinical settings including infections and malignancies. In human studies, AHCC® was orally administered at 3 g a day while most mouse studies used oral AHCC® in a range of 0.1-0.48 g/kg/day, except two studies where the doses were 1 and 3 g/kg/day, respectively. In the latter study, AHCC® was evaluated for colitis in mice. It is noticeable that 0.1-0.48 g/kg/day of AHCC® in mice is equivalent to 0.0081-0.039 g/kg/day of AHCC® in humans based on the guidance of the US FDA [38, 39]. These doses are similar to the recommended AHCC® doses of 1-3 g a day (0.017-0.05 g/kg/day based on weight 60 kg) for humans. The findings from these studies are summarized in the following sections (see also Table 1).

### 2.1. Infections

Influenza virus is one of the most significant viral infections that causes substantial mortality and morbidity in older adults, children, and immune-compromised hosts [40]. The effect of AHCC® on influenza viral infection has been studied, showing the possible beneficial effect, especially through affecting NK cells [24]. Supplementing mice orally with AHCC® (1 g/kg/day) improved survival and lung integrity upon intranasal challenge with influenza virus (H1N1) [24]. The mice that received AHCC® had increased NK cell percentages and activity as measured against YAC-1 target cells, along with decreased viral titers in the lungs [24]. The former finding could be a potential mechanism responsible for the beneficial effect of AHCC® in this mouse model in that NK cells were suggested to have a role in controlling influenza viral infection by secreting cytokines and expressing cytotoxic molecules [30]. The improvement of survival with enhanced viral clearance and NK cell lytic efficiency was also found in influenza virus-infected mice which were supplemented with a low-dose AHCC® (0.1 g/kg/day) [22]. Of note, a transient deficiency of NK and T cells was found in patients with severe H1N1 influenza [41]. Given the increased NK cells in mice treated with AHCC® [24], it would be intriguing to test whether AHCC® could increase NK cells in patients with H1N1 influenza. The effect of AHCC® can be beyond H1NI influenza. The survival benefit by AHCC® supplementation was observed in mice infected with avian (bird) influenza virus H5N1 which could infect humans and poultry although its mechanism is yet to be demonstrated [15]. In fact, the mortality rate of H5N1 avian influenza is much higher than that of past influenza pandemics, reaching up to 60% [42]. The available data support the implication of NK cells in controlling influenza virus via promoting the number and function of NK cells, raising the possible consideration of exploring the clinical utility of AHCC® for influenza viral infections, including avian influenza infection, in humans.

### 2.2. Malignancies

The immune system, which plays an essential role in the development and control of malignancies, can become tolerant to tumor cells by multiple mechanisms [36]. Different modalities such as cytokines and food supplements have been considered to boost NK cell immunity in treating cancers [3, 36]. Indeed, studies reported the possible beneficial effects of AHCC® supplementation in controlling cancers, especially in a combination with other anticancer therapies like chemotherapy [23]. NK cells appear to be involved in providing such effects. In an observational study without a placebo control, AHCC® supplementation (3 g/day) enhanced NK cell activity in a small number of patients with various cancers including the prostate [17]. Also, the possible role of AHCC® in suppressing the development of melanoma and immune mechanisms involved in this process was studied. In fact, antitumor immunity is critical in controlling melanoma as evidenced by the recent introduction of immunotherapies specifically enhancing T cell function through blocking inhibitory check point molecules expressed on T cells [43, 44]. In a mouse model of melanoma, AHCC® significantly delayed tumor development after B16-F0 melanoma inoculation [16]. This phenomenon was accompanied by an increase in the number of NK cells, tumor antigen-specific CD8^+^ T cells producing IFN-*γ* and gamma delta T cells [16]. The beneficial effect of AHCC® on murine B16 melanoma is further supported by a recent study reporting decreased melanoma sizes in mice supplemented with AHCC® with or without CpG-oligodeoxynucleotide (ODN), which is known to activate innate immunity and serve as an immunologic adjuvant [18]. The antitumor effects of low-dose 5-fluorouracil (5-FU) were potentiated by AHCC® in hepatoma 22 tumor-bearing mice through modulation of immune function, including increased percentages of NK cells [13]. However, no significant difference in NK cell activity was found between healthy human volunteers who took AHCC® (3 g/day × 4 weeks) and placebo, which could be related to small sample sizes (*n* = 10 and 11 for AHCC® and placebo groups, respectively) [26]. Given the potential effects of AHCC® on the number and function of NK cells that play an essential role in immune surveillance against malignancies, further human and animal studies on NK cell-mediated anticancer effects of AHCC® are warranted.

## 3. Effects of AHCC® on T Cell Immunity in Infections, Inflammations, and Malignancies

### 3.1. T Cell Immune Responses

T cells, a component of the adaptive immunity, play a critical role in defending hosts against microorganisms and malignancy [29]. CD4^+^ T cells are T helper (Th) cells with the capacity to promote the function of other immune cells such as B cells and macrophages by secreting cytokines and expressing costimulatory molecules [45, 46]. CD8^+^ T cells, which are cytotoxic T cells armed with the cytotoxic molecules perforin and granzymes, can kill infected or tumor cells [47]. Mushroom extracts, especially polysaccharides, are reported to promote immune responses to tumor by affecting the functions of T cells and other immune cells [3]. Oligosaccharides are the most abundant component of AHCC® accounting for about 74% of its dry weight [6] [7]. Indeed, the effects of AHCC® on T cell immunity are observed in humans and animals (see Table 2 for summaries). In human studies, AHCC® was orally administered at 3 g a day (0.05 g/kg/day based on 60 kg weight), while most mouse studies used oral AHCC® in a range of 0.36-1 g/kg/day, which is equivalent to 0.029-0.081 g/kg/day of AHCC® for humans based on the guidance of the US FDA [38, 39]. In an observational study of healthy adults aged 50 or older, AHCC® supplementation (3 g/day for 60 days) increased the frequency of peripheral CD4^+^ and CD8^+^ T cells producing IFN-*γ* and/or TNF-*α* at 30 and 60 days of ELCM supplementation compared to the baseline [28]. Such a finding was still noticed at 30 days after discontinuing AHCC®. However, additional studies are necessary to determine the effect of AHCC® on other T cell functions. The effects of AHCC® on T cells could be mediated by affecting innate immune cells since oligosaccharides including *α*-glucans and *β*-glucans are known to stimulate innate immune cells such as monocytes, macrophages, and dendritic cells that can modulate the activation and differentiation of T cells [5, 48–51]. We recently reported the promotion of Th 1 and 17 cells, which predominantly produced IFN-*γ* and IL-17, respectively, by AHCC® through inducing IL-1*β* production from monocytes in humans [20]. In accordance with this finding, the culture supernatants of AHCC®-treated murine monocytic J744.2 cells promoted the production of TNF-*α* from splenic T cells of mice [12] while AHCC® induced IL-8 production from human myelocytic THP-1 cells via activating mitogen-activated protein kinases (MAPKs) and NF-*κ*B pathways [14]. In a mouse study, AHCC® administration increased cytokine production in the intestine fluid dependently of TLR2 and TLR4, suggesting the implication of these molecules in AHCC®-mediated immune modulation [52]. Also, an increase in the number of circulating dendritic cells (DCs) was found in healthy adults after receiving AHCC® (3 g/day × 4 weeks), suggesting the possible implication of AHCC® in promoting immune responses via modulating DCs [26]. Overall, the data support that AHCC® can modify T cell immunity in part by activating innate immune cells with the capacity to promote T cell activation.

### 3.2. Infections, Inflammations, and Malignancies

The possible effects of AHCC® on T cell immunity may have biological significance in developing immune responses to antigens. This is evidenced by a study reporting increased NKT cells and CD8^+^ T cells along with increased protective antibody titers to influenza B in healthy people who received influenza vaccine and AHCC® supplementation (3 g/day × 3 weeks) [25]. Also, in a mouse model of West Nile encephalitis, mice supplemented with AHCC® (600 mg/kg every other day) had the expansion of *γδ*T cells, which had an important role in controlling West Nile virus infection, along with decreased viremia [27]. The potential beneficial effects of AHCC® on the immune system and bacterial infection were previously reported in the hindlimb unloading mouse model of space flight conditions which could adversely affect the immune system [9, 10, 53]. Indeed, a recent study using the same mouse model showed a trend towards increased T cell proliferation in mice supplemented with AHCC® compared to control mice [54].

The effect of AHCC® on CD4^+^ T cells was found in hepatoma 22 tumor-bearing mice [13]. Compared to mice treated with 5-FU, mice treated with 5-FU and AHCC® had an increase in the percentage of CD4^+^ T cells and levels of IL-2, the T cell growth factor, in peripheral blood [13]. This observation raises the possibility that the antitumor effect of AHCC® can be mediated in part by modulating T cell function. The immune system, including T cells, can become tolerant to tumor cells by multiple mechanisms [36]. Probably, the best-known mechanism is suppressing T cell activation and effector function by triggering inhibitory checkpoint molecules, including CTLA-4 (cytotoxic T lymphocyte-associated antigen 4) and PD-1 (programmed cell death protein 1), expressed on T cells [43, 44]. Immunotherapy targeting these molecules has made substantial impacts in oncology by improving the survival of patients with cancers such as non-small cell lung cancer, bladder cancer, and melanoma [32, 44]. However, the effects of mushroom extracts on inhibitory checkpoint molecules in T cells are largely unknown.

Although the exact mechanism of how AHCC® affects T cells is yet to be demonstrated, AHCC® can promote T cell function through activating innate immune cells especially with oligosaccharides like other mushroom extracts [3, 12, 14, 20, 26]. A recent study reported that AHCC® supplementation (75 mg/day) improved lymphocyte-driven colitis in recombination activating gene 1- (RAG-1-) deficient mice transferred with CD4^+^CD62L^+^ T cells [21]. In this study, the production of IL-6, IL-17, and IL-10 by mesenteric lymph node cells as well as STAT4 phosphorylation in splenic CD4^+^ T cells were decreased in colitis mice supplemented with AHCC®. Of note, we noticed the suppression of cytokine production from human CD4^+^ T cells activated with anti-CD3 and CD28 antibodies *in vitro* in the presence of AHCC® (Kang et al., unpublished data). This suppressive effect of AHCC® appeared to be greater on IL-10, IL-17, and IFN-*γ*. However, AHCC® did not affect the proliferation and survival of human CD4^+^ T cells activated with anti-CD3 and CD28 antibodies. We also determined the effect of AHCC® on the transcription factor FOXP3 that is highly upregulated in T cells with regulatory function [29]. No effect of AHCC® on FOXP3 expression in human CD4^+^ T cells was noticed (Kang et al., unpublished data). As aforementioned, since AHCC® can promote T cell function by activating innate immune cells, it is possible that the effects of AHCC® on T cells could depend on the context of immune activation. For instance, AHCC® could directly suppress cytokine production from activated T cells while the function of T cells may be promoted by innate immune cells in the presence of AHCC®.

## 4. Conclusions

AHCC®, which is an extract from the culture of shiitake (*Lentinula edodes*) mycelia, has a broad range of effects on the immune system including NK and T cells. Such effects could be executed by directly modulating the numbers and functions of these cells as well as by affecting the function of monocytes, macrophages, and DCs with the capacity to promote T cell function. Plus, the effects of AHCC® on NK and T cells appear to have biological implications as suggested by the results of clinical studies and *in vivo* animal studies on infections, inflammations, and tumors. Studies exploring additional immunologic effects of AHCC® and mechanisms underlying these effects in health and disease are warranted. Of note, the intestinal microbiome has been a subject of intensive investigations in the field of food supplements and medicinal foods including extracts of mushrooms. However, the effects of AHCC® on the intestinal microbiome is unknown. The results of such studies exploring the effects of AHCC® on the immune system and/or microbiome would lead to advancing our understanding in the utility of medical mushrooms including AHCC®, especially in the context of recently introduced immunotherapies targeting inhibitory checkpoint molecules including CTLA-4 and PD-1.

## Figures and Tables

**Table 1:** The effects of AHCC® on natural killer (NK) cells in health and disease.

Host	Condition	AHCC® supplementation dose	Effects	Reference
Mice	Influenza viral infection (H1N1)	^∗^Oral, 1 g/kg/day ^∗∗^(25 mg/day)	Increased NK cell percentage and activity Improved survival, lung integrity, and viral titers	[24]
Mice	Influenza viral infection (H1N1)	^∗^Oral, 0.1 g/kg/day ^∗∗^(2.5 mg/day)	Increased NK cell lytic efficiency Improved survival with enhanced viral clearance	[22]
Mice	Melanoma	^∗^Oral, 12 mg/day ^∗∗^(0.48 g/kg/day)	Increased NK cell number Increased *γδ*T cell number Increased tumor antigen-specific CD8^+^ T cells producing IFN-*γ* Delayed melanoma development	[16]
Mice	Melanoma	^∗^Oral, 10 mg/day with or without i.p. CpG ODN ^∗∗^(0.40 gm/kg/day)	Decreased melanoma development (size) in mice treated with AHCC® alone or AHCC® and CpG ODN No immune cells analyzed	[18]
Mice	Hepatoma	^∗^Oral, 0.36 g/kg/day With 5-FU ^∗∗^(9 mg/day)	Increased NK cell percentage Increased CD4^+^ T cell percentage Potentiate the effect of 5-FU on tumor weight, size, and by AHCC®	[13]
Humans	Cancers (open label observational)	Oral, 3 g/day	Enhanced NK cell activity	[17]
Humans	Healthy volunteers: a double-blind, placebo-controlled	Oral, 3 g/day or placebo	No difference in NK cell activity between AHCC® and placebo groups	[26]

^∗^Dose used in each study. ^∗∗^Dose in g/kg/day was converted to dose in mg/day or vice-versa based on mouse weight of 25 g.

**Table 2:** The effects of AHCC® on T cells in health and disease.

Host or origin of cells	Condition	AHCC® supplementation dose	Effects	Reference
Mice	*In vitro*	100 *μ*g/mL	Promoted the production of TNF-*α* by splenic T cells by inducing IL-1 from murine monocytic J744.2 cells	[12]
Mice	West Nile virus infection in young and old mice	^∗^Oral, 0.6 g/kg every other day ^∗∗^(15 mg every other day)	Increased *γδ*T cells Decreased viremia Decreased mortality in young but not old mice	[27]
Mice	Hepatoma	^∗^Oral, 0.36 g/kg/day With 5-FU ^∗∗^(9 mg/day)	Increased CD4^+^ T cell percentage and circulatory IL-2 levels Potentiate the effect of 5-FU on tumor weight, size, and by AHCC®	[13]
Mice	A hindlimb unloading mouse model of space flight conditions	^∗^Oral, 1 g/kg/day ^∗∗^(25 mg/day)	Trend towards increased T cell proliferation not reaching the level of statistical significance	[54]
Mice	Lymphocyte-driven colitis model	^∗^Oral, 75 mg/day ^∗∗^(3 g/kg/day)	Decreased STAT4 phosphorylation in splenic CD4^+^ T cells Decreased colitis	[21]
Humans	Healthy volunteers age 50 or older	Oral, 3 g/day	Increased frequency of CD4^+^ and CD8^+^ T cells producing IFN-*γ* and/or TNF-*α*	[28]
Humans	*In vitro*	500 *μ*g/ml	Promoted the production of IFN-*γ* and IL-17 by CD4^+^ T cells by inducing IL-1*β* production from monocytes	[20]
Humans	Healthy adults receiving influenza vaccination	Oral, 3 g/day	Increased CD8^+^ T cells Increased NKT cells Increased protective antibody titers to influenza B strain after influenza vaccination	[25]
Humans	*In vitro*	250-500 *μ*g/ml	Decreased IL-10, IL-17, and IFN-*γ* production from purified CD4^+^ T cells stimulated with anti-CD3 and CD28 antibodies No effect on proliferation and survival No change in FOXP3 expression	Kang et al., unpublished observations

^∗^Dose used in each study. ^∗∗^Dose in g/kg/day was converted to dose in mg/day or vice-versa based on mouse weight of 25 g.
